# Maternal Betaine Supplementation throughout Gestation and Lactation Modifies Hepatic Cholesterol Metabolic Genes in Weaning Piglets via AMPK/LXR-Mediated Pathway and Histone Modification

**DOI:** 10.3390/nu8100646

**Published:** 2016-10-18

**Authors:** Demin Cai, Mengjie Yuan, Haoyu Liu, Shifeng Pan, Wenqiang Ma, Jian Hong, Ruqian Zhao

**Affiliations:** 1 Key Laboratory of Animal Physiology & Biochemistry, Nanjing Agricultural University, Nanjing 210095, China; yzu.jsnj.ice@163.com (D.C.); senrutoday@163.com (M.Y.); pan.sf@163.com (S.P.); wq8110@126.com (W.M.); jian_hong602@sina.com (J.H.); 2 Department of Medical Cell Biology, Uppsala University, Uppsala SE-75123, Sweden; haoyu.liu@mcb.uu.se; 3 Jiangsu Collaborative Innovation Center of Meat Production and Processing, Quality and Safety Control, Nanjing 210095, China

**Keywords:** betaine, AMPK, LXR, cholesterol metabolism, maternal diet, piglets

## Abstract

Betaine serves as an animal and human nutrient which has been heavily investigated in glucose and lipid metabolic regulation, yet the underlying mechanisms are still elusive. In this study, feeding sows with betaine-supplemented diets during pregnancy and lactation increased cholesterol content and low-density lipoprotein receptor (LDLR) and scavenger receptor class B type I (SR-BI) gene expression, but decreasing bile acids content and cholesterol-7a-hydroxylase (CYP7a1) expression in the liver of weaning piglets. This was associated with the significantly elevated serum betaine and methionine levels and hepatic *S*-adenosylmethionine (SAM) and *S*-adenosylhomocysteine (SAH) content. Concurrently, the hepatic nuclear transcription factor liver X receptor LXR was downregulated along with activated signal protein AMP-activated protein kinase (AMPK). Moreover, a chromatin immunoprecipitation assay showed lower LXR binding on CYP7a1 gene promoter and more enriched activation histone marker H3K4me3 on LDLR and SR-BI promoters. These results suggest that gestational and lactational betaine supplementation modulates hepatic gene expression involved in cholesterol metabolism via an AMPK/LXR pathway and histone modification in the weaning offspring.

## 1. Introduction

Betaine, also called TMG (trimethylglycine), is a natural component existing in all living organisms which can be obtained from diet or be synthesized from choline in mammals [1]. Both clinical investigations and experimental studies demonstrate its vital function of hepatoprotection in liver metabolic diseases, including nonalcoholic liver disease and bile acid-induced liver injury [2,3,4]. Recent studies have shown that hepatic lipid metabolic abnormality in the postnatal period is tightly linked to higher risk of later development of chronic metabolic diseases [5]. Therefore, whether betaine is a prophylactic strategy to decrease the susceptibility to metabolic illnesses in adulthood through controlling cholesterol metabolism in the liver during early life remains to be clarified.

Hepatic cholesterol homeostasis is maintained by series of biochemical reactions including cholesterol synthesis, uptake, and efflux [6]. HMGCR (3-hydroxy-3-methylglutaryl-CoA reductase) is the primary enzyme regulating cholesterol biosynthesis [7]. During transportation, excessive hepatic cholesterol is transported into the serum via low-density lipoprotein receptor (LDLR) [8] whilst the reverse transportation depends on scavenger receptor class B type I (SR-BI) [9]. Furthermore, the conversion from cholesterol to bile acid is carried out by cholesterol-7α-hydroxylase (CYP7a1) and cholesterol-27a-hydroxylase (CYP27a1) [10]. Because of the critical action of nuclear receptors (NRs) on the regulation of metabolic genes, cholesterol metabolic genes expressions are shown to be modulated by NRs in numerous literatures [11,12,13]. Liver X receptor (LXR) plays a crucial role in the regulation of cholesterol efflux and influx by inhibiting the LDLR pathway or reducing the expression of SR-BI [14,15]. LXR is also found to bind on the promoter of CYP7a1 gene [16] and then controls hepatic bile acids formation directly or interacts with peroxisome proliferator-activated receptor alpha (PPARα) [17]. In contrast, farnesoid X receptor (FXR) has been well demonstrated to be a bile acid sensor and predominant regulator of the CYP7a1 gene [18,19]. However, it is still elusive whether nuclear receptors regulate the hepatic cholesterol metabolic gene in vivo during the early stage of life.

Betaine participating in epigenetic regulation for gene expression has been documented in a number of studies, and is correlated with methionine metabolism [1]. In this metabolic cycle, methionine, in turn, is converted to *S*-adenosylmethionine (SAM), *S*-adenosylhomocysteine (SAH) and homocysteine using enzymes methionine adenosyl transferase (MAT), glycine N-methyltransferase (GNMT), and S-adenosylhomocysteine hydrolase (AHCY), respectively [20]. Finally, homocysteine is remethylated to methionine catalyzed by betaine–homocysteine methyltransferase (BHMT) to complete this metabolic cycle [21]. Notably, SAM is critical for epigenetic regulation because it supports methyl groups for DNA and protein methylation. Especially, in the liver, the ratio of SAM to SAH is a pivotal indicator to assess the methylated status of global and specific genes [22]. It is worthwhile to mention that AMP-activated protein kinase (AMPK), a SAM sensor, is able to bind SAM directly [21] and is a kinase upstream in the LXR pathway, so can inactivate LXR-mediated gene transcription [23]. Moreover, Dahlhoff et al. provides the evidence that methyl donors, including betaine and methionine supplementation, enhance SAM formation and then activate AMPK-signaling pathway in mice [24].

Therefore, we hypothesized that maternal dietary supplementation of betaine may affect cholesterol metabolic genes through epigenetic mechanisms and nuclear-receptor-mediated signal pathways in the liver of offspring. It would be interesting to eventually use betaine supplementation as a prophylactic strategy to decrease the susceptibility to metabolic illnesses in adulthood.

## 2. Materials and Methods

### 2.1. Animals and Sampling

Sixteen cross-bred Landrace X Yorkshire sows (second parity) were inseminated artificially with a mixture of Duroc semen samples obtained from two littermate boars when estrus occurred. One week later, 8 sows were randomly assigned to the treated group (Bet), and the remaining 8 were assigned to the control group (Con). Sows in control group received basal diet whilst betaine group were fed betaine-supplemented (3 g/kg) diet during gestation and lactation (98% pure betaine hydrochlorides; SKYSTONE FEED CO., Ltd., Yixing. Jiangsu, China). The diet composition is shown in Table 1.

The housing barn had a controlled environmental system with constant temperature at 25 °C and 50% humidity on a 12 h/12 h light/dark cycle. Feed was offered at 05:00, 10:00, and 17:00 per day with free access to water. Twenty-four hours after farrowing, litter size was adjusted to 7–8 piglets per litter in the same group. After 35 days of age, one piglet per litter close to the mean body weight of the litter was killed for sampling. Serum samples were collected immediately and stored at −80 °C and liver samples (no gall bladder) were taken within 20 min postmortem then snap-frozen in liquid nitrogen and stored at −80 °C for further analysis.

The animal experiment was undertaken with the project number 2012CB124703, Animal Ethics Committee of Nanjing Agricultural University, following the “Guidelines on Ethical Treatment of Experimental Animals’’ (2006) No. 398 set by the Ministry of Science and Technology, China.

### 2.2. Measurement of Cholesterol and Bile Acid Concentrations

The content of total cholesterol and triglycerides (TG) in serum was measured using commercial assay kit (E1005 and E1003 respectively; Applygen Technologies, Inc. Beijing, China). Concentrations of LDL cholesterol (LDLC) and high-density lipoprotein cholesterol (HDLC) in serum were measured with respective assay kits (006340 and 006328, Beijing BHKT Clinical Reagent Co., Ltd., Beijing, China). The cholesterol and TG in liver was determined according to the instruction of a tissue commercial assay kit (E1015 and E1013 respectively; Applygen Technologies, Inc. Beijing, China). Hepatic and serum bile acid content was detected by enzymatic colorimetric methods according to a commercial bile acid assay kit (E003; Nanjing Jiancheng Bioengineering Institute, Nanjing, Jiangsu, China).

### 2.3. Determination of Serum Betaine Content and Hepatic S-Adenosyl Methionine and S-Adenosyl Homocysteine Concentrations

Serum betaine concentrations were analyzed in China National Feed Quality Control Center, Chinese Academy of Agricultural Sciences, Beijing, China by liquid chromatography (Aglient 1200, Aglient Techologies. Santa Clara, California, USA)—MS (API 5000TM; AB Foster City, California, USA) system. Hepatic SAM and SAH concentrations were determined by Quantitative Porcine Competitive ELISA kits (S200FC and S198FC respectively; Hermes Criterion Biotechnology, Vancouver, BC, Canada) following the manufacturer’s protocol.

### 2.4. Serum Hormones and Amino Acids Profile

Serum concentrations of insulin, glucagon, and cortisol were measured with respective commercial RIA kits (No. F01PZB, F03PZB and D10PZB Beijing North Institute of Biological Technology, Beijing, China) with 10% and 15% of intra- and inter-assay variations.

Serum samples for measuring the free amino acid concentration were prepared according to a previous publication [21]. Serum concentrations of free amino acids were determined with an automatic amino acid analyzer (L-8900, Hitachi, Japan) in duplicate. The intra- and inter-assay coefficients of variation were 3% and 6%, respectively.

### 2.5. Real-Time PCR for mRNA Quantification

Total RNA was extracted from the liver samples (200 mg). The frozen tissue sections were homogenized in TRIzol Reagent (Invitrogen, Santa Rosa, USA) according to the manufacturer’s protocol. Approximately 2 μg RNA was reverse transcribed into cDNA with the random hexamer primers (Promega, Madison USA) and reverse transcription products were stored at −20 °C. The quantitative analysis of gene expression was carried out on an Mx3000P (Stratagene, Santa Rosa, USA) real-time PCR system with 2 µL diluted cDNA (1:25). Stable reference gene peptidylprolyl isomerase A (PPIA) was selected for the normalization of target gene expression level. The final result was expressed as relative expression by comparing the amount of target gene to PPIA. All primers were synthesized by Generay Biotech and listed in Table 2.

### 2.6. Western Blotting for Protein Quantification

Total protein was extracted from 200 mg frozen liver sample as described before. Briefly, samples were homogenized with RIPA buffer (No. 89900, Thermo, Santa Rosa, USA), and incubated for 20 min on ice, followed by centrifugation for 10 min at 12,000 rpm at 4 °C. The protein content of each sample was quantified with a Pierce BCA Protein Assay kit (No. 23225, Thermo, Santa Rosa, USA). Western blot analysis for target protein was carried out depending on recommended instruments provided by the manufacturers. β-Actin and GAPDH (glyceraldehyde-3-phosphate dehydrogenase) were used as references for total protein, while H1 was used as a reference for nuclear protein in the Western blot analysis. All antibodies used are listed in Table 3.

### 2.7. Chromatin Immunoprecipitation

Chromatin immunoprecipitation (ChIP) analysis was performed according to our previous publication [25]. Crude chromatin preparations were isolated from approximately 200 mg frozen liver samples and were sonicated and precleared with salmon sperm DNA-treated protein G agarose beads (40 μL, 50% slurry, sc-2003, Santa Cruz Biotechnology, Santa Cruz, USA). The precleared chromatin products mixed with 2 μg of specific primary antibody (H3, ab1791, Abcam, Cambridge, USA; H3K4me3, ab8580, Abcam; H3K27me3, 17-622, Millipore, Darmstadt, Germany) overnight at 4 °C. We used normal rat IgG as a negative control. For capturing the immunoprecipitated chromatin complexes, we added protein G agarose beads again into the aforementioned mixture. Finally, we released DNA fragments from reverse cross-linking and quantified target gene fragments using with specific primers (Table 2).

### 2.8. Statistical Analysis

All data are presented as means ± S.E.M. and were analyzed using independent two-tailed Student’s *t*-test with SPSS 19.0 for Windows (SPSS, Inc. Armonk, USA). Since none of the detected parameters showed sex disparity, we pooled male and female together. Values of mRNA and protein are presented as the fold change relative to the mean value of the control group. For all analyses, *p* < 0.05 was considered significant.

## 3. Results

### 3.1. Serum Concentrations of Metabolites in Weaning Piglets

As shown in Table 4, maternal betaine supplementation did not change body weight or liver weight in the weaning piglets. However, serum levels of betaine and LDLC/HDLC, together with methionine and phenylalanine, were found to be higher in the betaine-exposed piglets compared to that in the control group (*p* < 0.05).

### 3.2. Cholesterol Metabolism in Weaning Piglets

**Figure 1:**
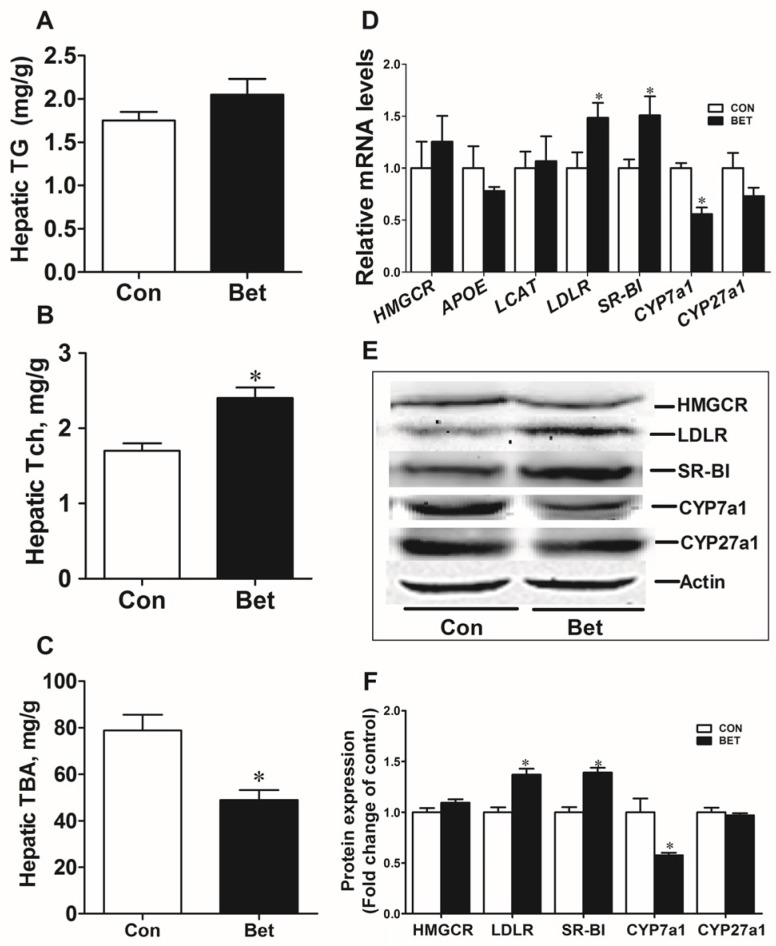
Hepatic triglycerides (**A**); total cholesterol (**B**); and total bile acids (**C**) of the offspring piglets at weaning; hepatic mRNA level (**D**); Western blotting analysis and graphic summary (**E**,**F**) of cholesterol metabolic genes in the liver of weaning piglets. Values are means ± SEM, *n* = 8. Different from control, * *p* < 0.05.

Maternal betaine supplementation significantly increased hepatic cholesterol (Figure 1B) (*p* < 0.05) and decreased bile acids content (Figure 1C) (*p* < 0.05), which is associated with upregulated LDLR and SR-BI expression, and downregulated CYP7a1 expression, both at mRNA and protein level in the weaning piglets (Figure 1D–F, *p* < 0.05).

### 3.3. Methionine Metabolism in Weaning Piglets

**Figure 2:**
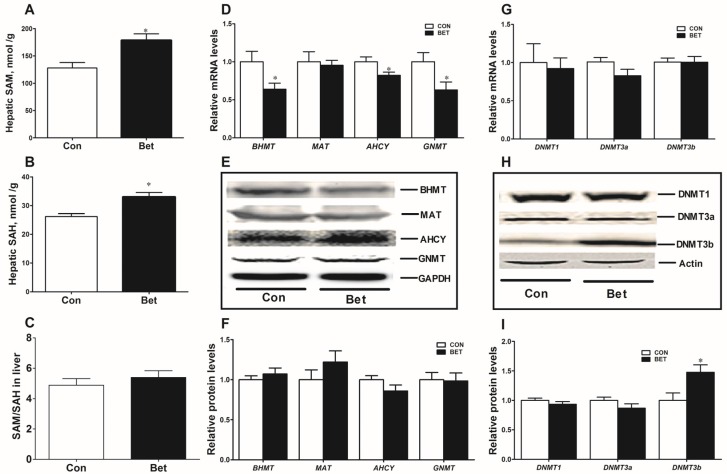
Concentration of hepatic *S*-adenosylmethionine (SAM) (**A**) and *S*-adenosylhomocysteine (SAH) (**B**); as well as the ratio of hepatic SAM to SAH (**C**) in weaning piglets; hepatic mRNA abundance (**D**); and Western blotting analysis plus graphic summary (**E**, **F**) of proteins expression involved in methionine metabolic cycle; DNA methyltransferases including DNMT1, DNMT3a, and DNMT3b expression at mRNA (**G**) and protein level (**H**, **I**) in the liver of weaning piglets. Values are means ± SEM, *n* = 8. Different from control, * *p* < 0.05.

Maternal betaine supplementation significantly increased hepatic SAM (Figure 2A, *p* < 0.05) and SAH content (Figure 2B, *p* < 0.05), yet the ratio of SAM to SAH was not altered in piglets (Figure 2C). Although the key genes involved in methionine metabolism including BHMT, AHCY, and GNMT were downregulated at transcriptional level (Figure 2D, *p* < 0.05), the protein expression of these enzymes was not changed (Figure 2E,F). Moreover, among these key methyltransferases, only DNA methyltransferase 3b (DNMT3b) was upregulated at protein level (Figure 2H,I, *p* < 0.05), while DNMT1 and DNMT3a expressions were not changed either at mRNA (Figure 2G) or protein level (Figure 2H,I) in the liver of betaine-exposed piglets.

### 3.4. Transcriptional Regulation of Cholesterol Metabolic Genes

**Figure 3:**
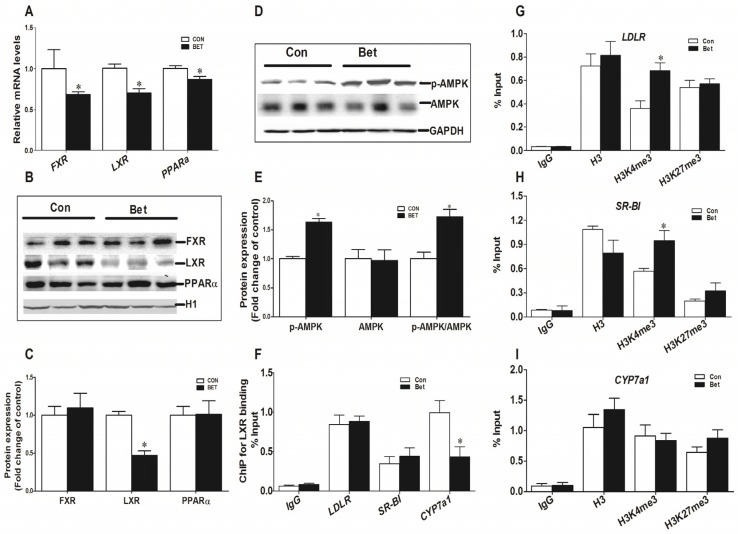
Hepatic mRNA abundance (**A**) and Western blotting analysis (**B**, **C**) of nuclear transcriptional factors farnesoid X receptor (FXR), liver X receptor (LXR), and peroxisome proliferator-activated receptor alpha (PPARα); (**D**, **E**) illustrates Western blotting analysis and graphic summary of signal protein AMP-activated protein kinase (AMPK) in the liver of weaning piglets. Chromatin immunoprecipitation (ChIP) analysis of LXR binding on low-density lipoprotein receptor (LDLR), scavenger receptor class B type I (SR-BI), and cholesterol-7α-hydroxylase (CYP7a1) genes’ promoters (**F**); and respective ChIP analysis of histone modifications on *LDLR* (**G**); *SR-BI* (**H**); and *CYP7a1* (**I**) genes’ promoters in the liver of weaning piglets. Values are means ± SEM, *n* = 8. Different from control, * *p* < 0.05.

Maternal betaine supplementation significantly reduced the mRNA level of hepatic nuclear transcription factors *FXR, LXR*, and *PPARα* (Figure 3A, *p* < 0.05), but only LXR protein content was downregulated (Figure 3B,C, *p* < 0.05) in parallel with its mRNA. Phosphorylated-AMPK was found to be increased significantly at the protein level (Figure 3D,E, *p* < 0.05). The ChIP assay was used to detect the LXR binding enrichment and histone modification on the changed cholesterol metabolic genes promoter. As illustrated in Figure 3F, the betaine-exposed piglets demonstrated lower LXR binding to the CYP7a1 gene promoter (*p* < 0.05), and significantly enriched histone activation marker, H3K4me3, on *LDLR* and *SR-BI* genes’ promoters (Figure 3G,H, *p* < 0.05).

## 4. Discussion

It has been shown that mother-derived betaine can be accumulated in the fetus at a higher concentration than that in maternal circulation [26]. Moreover, dietary betaine supplementation in lactational sows significantly increases betaine content in the milk [27]. Here, we found that maternal betaine supplementation during gestation and lactation caused significant increase in serum betaine levels of the weaning offspring. In mammals, methionine is synthesized from the homocysteine using betaine as a substrate [28]. Serum methionine level is found to be increased following dietary betaine supplementation in human subjects [29]. In agreement with these findings, we detected elevated methionine concentration associated with higher betaine level in the serum of betaine-exposed piglets.

The effects of betaine on cholesterol homeostasis are controversial. Betaine supplementation in human subjects increased serum total cholesterol and LDLC concentrations [30,31]. On the contrary, dietary betaine supplementation was also reported to decrease the serum total cholesterol concentration in pigs [32]. A negative correlation between plasma betaine concentration and plasma concentrations of total cholesterol and LDLC was reported in an epidemiological research [33]. In the present study, betaine-exposed weaning offspring had 41.2% higher hepatic cholesterol and 62.5% lower hepatic bile acids content along with a significantly elevated serum LDLC/HDLC compared with the control groups. We suggest this is partly due to the reduced cholesterol transformation and increased cholesterol transportation.

In line with Hu et al. finding that in vivo injection with betaine significantly increases hepatic cholesterol content and decreases hepatic CYP7a1 expression in newly hatched chicks [34], herein, piglets demonstrated obviously reduced hepatic CYP7a1 transcriptional and translational expression under maternal betaine supply. In this process, *CYP7a1* transcription is predominantly regulated by the nuclear receptor FXR, which induces the small heterodimer partner (SHP) in liver that downregulates CYP7a1 expression [35].

LXR has been regarded as a dominant regulator of *CYP7a1* transcription by Gupta et al. [36]. We provided the first evidence that LXR directly binds to porcine *CYP7a1* gene and positively regulates *CYP7a1* transcription by ChIP assay. Although betaine functions on LXR are still unknown, betaine promotes SAM formation by increasing methionine level through one-carbon metabolism [37]. It is reported that AMPK may bind SAM directly and acts as a direct SAM-sensor, thus methyl donation, including betaine and methionine supplementation, result in AMPK signaling pathway activation [24]. Again, AMPK has been demonstrated to be a kinase upstream of LXR, and the activated AMPK (phospho-AMPK) inhibits endogenous LXR ligand production, which then diminishes LXR expression and blocks LXR transcriptional regulation [23]. Thus the lower LXR expression and binding on *CYP7a1* may, at least, be attributed to the activated AMPK and elevated SAM content in the liver of betaine-exposed piglets at weaning.

Methylation of DNA and chromatin histones by specific methyltransferases utilize SAM as the methyl donor [38] and hepatic SAM/SAH ratio is a critical indicator of cellular methylation reactions [22]. In our current data, hepatic SAM was significantly increased in piglets of betaine group, but the ratio of SAM to SAH had not changed. Similar to a preceding finding of Medici et al. [39], no significant alteration was observed for the expression of key enzymes involved in methionine metabolism or the methyltransferases. Thus, we excluded DNA methylation analysis for these changed cholesterol metabolic genes. However, we found more enrichment of histone-activated marker H3K4me3 on the *LDLR* and *SR-BI* genes’ promoters, which is associated with enhanced gene transcription. It is noted that H3K4me3 is trimethylated by a SAM-dependent enzyme, SETD7 [40], and our previous study has demonstrated that maternal betaine diet increases SETD7 expression in the liver of offspring [41]. Therefore, in the present study, enhanced SAM content may greatly contribute to activate histone modification in the promoters of hepatic cholesterol metabolic genes in the betaine-exposed weaning piglets.

It is worthwhile to mention that methionine–homocysteine cycle is strongly regulated by feedback mechanisms [42]. SAM concentrations are maintained by stimulating the BHMT pathway [43]. Betaine activates BHMT for methionine and SAM formation [44,45]; in contrast, SAM suppresses BHMT expression in order to decrease utilization of betaine as a methyl donor [42,46]. Betaine supplementation in maternal diet downregulated hepatic BHMT gene expression in the weaning piglets, much like a negative feedback loop.

Age-dependent effects perform a critical function on gene expression in the studies of maternal nutritional programming, leading to the varied profiles of gene regulation at different life stages [47,48,49]. Our studies also showed the different expressions of hepatic genes involved in cholesterol metabolism between newborn [50] and weaning stage of the piglets exposed to maternal betaine diet.

In summary, our findings indicate that maternal dietary betaine supplementation during gestation and lactation modifies hepatic cholesterol metabolic gene expression in weaning piglets through AMPK/LXR-dependent pathway and histone modifications. Changes in hepatic cholesterol metabolism in weaning offspring may carry on to adulthood, causing life-time consequences in cholesterol homeostasis later in adult life, and this should be addressed in future studies.

## Figures and Tables

**Table 1:** Composition and nutrient content of the experimental diet.

	Gestation		Lactation	
	Con	Bet	Con	Bet
Ingredient, g/kg				
Corn	370	370	332.5	332.5
Wheat	300	300	100	100
Bran	80	80	50	50
Soybean meal	170	170	253	253
Maize starch	0	0	150	150
Lignocelluloses	30	30	0	0
CaHPO4	20	20	20	20
Soybean oil	8	8	34.5	34.5
Fish meal	0	0	40	40
Premix *	20	20	20	20
Betaine	0	3	0	3
Digestible energy, MJ/kg	13.1	13.1	14.39	14.39
Calculated composition %				
Crude protein, %	15	15	18	18
Crude fiber, %	4.5	4.5	2.3	2.3
Calcium, %	0.84	0.84	0.9	0.9
Phosphorous, %	0.65	0.65	0.7	0.7

* The premix contains (per kilogram): Vitamin A: 240,000 IU; vitamin D-3: 60,000 IU; vitamin E: 720 IU; vitamin K-3: 30 mg; vitamin B-1: 30 mg; vitamin B-2: 120 mg; vitamin B-6: 60 mg; vitamin B-12: 360 mg; niacin: 600 mg; pantothenic acid: 300 mg; folic acid: 6 mg; manganese sulphate: 1.0 g; zinc oxide: 2.5 g; ferrous sulphate: 4.0 g; copper sulphate: 4.0 g; sodium selenite: 6 mg; calcium: 150 g; phosphorus: 15 g; sodium chloride: 40 g.

**Table 2:** Nucleotide sequences of specific primers.

Target Genes	Sequences (5′ to 3′)		GenBank No.
mRNA expression			
*AHCY*	F: gtggtggtgtgtggctacgg	R: gcagagcacagatggggtca	NM_001201381.1
*APOE*	F: gtgcgcaaccgcttggtgctct	R: gacgagccgcttgcgcacgtt	NM_214308.1
*CYP7a1*	F: tagcaggcttcccgattc	R: ctgaccagttccgagatgtg	AK230868.1
*CYP27a1*	F: tgtggctcgcatcgttc	R: tcacctggcagctcctt	EF625352.1
*GNMT*	F: acaaagatgtgccca	R: gtgctgaggatgtggtcgta	NM_001110419.2
*MAT*	F:ctgacagtcctgtcttgggagc	R: gccagagtgattctttgatgcc	NC_010458.3
*BHMT*	F:gaggctgtgtgggcagttgaag	R: acaatggatgctcctgcctttacc	NM_001200042.1
*DNMT1*	F: tcagggaccacactgtaag	R: gctgcagccattcttcttgt	DQ060156.1
*DNMT3a*	F: ggctcttctttgagttctaccg	R: gcgagatgtccctcttgtca	DQ785811.1
*DNMT3b*	F: tgaagagtccatcgctgttg	R: caatcaccaggtcaaaggg	NM_001162404.1
*FXR*	F: cggagaagcattacca	R: aagcattcagccaaca	XM 001928800.2
*HMGCR*	F: caggctgaagtaagggaga	R: cacgaagtaggtggcga	DQ432054.1
*LDLR*	F: actgctcatcctcc tctt	R: ttccgtggtcttctggta	AF065990.1
*LXR*	F: atttccaggagtgccgtctt	R: cttgccgcttcagtttctt	AB254406.1
*LCAT*	F: ggctggtggaagaaatgc	R: gggttggcgtagtaagaaata	NM_001164856.1
*PPARα*	F:actgaacatcgaatgtagaatct	R: tctgaatcttacagctccgatc	NM_001044526.1
*PPIA*	F: gactgagtggttggatgg	R: tgatcttcttgctggtctt	NC_010460.3
*SR-BI*	F:tcaagcagcaggtcctcaag	R: cttgtgcctgaactccctgta	NM_213967.1
ChIP assay			
*LDLR* fragment	F: tcagaggagaggaagtggct	R: atccagcgctcagatgaat	
*SR-BI* fragment	F: gttgcatgaatgagcctact	R: cgtgaattccataggtaaca	
*CYP7a1* fragment	F: tgtctccacgggcgtaccaga	R: gtggcaatatacagacatct	

**Table 3:** Antibodies for this experiment.

Antibodies	MW	Species	Source	Catalogue No.
Western Blotting				
GNMT	33 kd	Rabbit	proteintech^TM^	18790-1-AP
BHMT	50 kd	Rabbit	proteintech^TM^	15965-1-AP
MAT	38 kd	Rabbit	proteintech^TM^	15952-1-AP
AHCY	60 kd	Rabbit	proteintech^TM^	10658-3-AP
DNMT1	184 kd	Rabbit	Santa Cruz	sc-20701
DNMT3a	102 kd	Rabbit	Bioworld	BS6587
DNMT3b	96 kd	Rabbit	Bioworld	BS2572
HMGCR	97 kd	Rabbit	Bioworld	BS6625
LDLR	160 kd	Rabbit	proteintech^TM^	10785-1-AP
SR-BI	82 kd	Rabbit	Abcam	ab137829
CYP7a1	57 kd	Rabbit	Abcam	ab79847
CYP27a1	60 kd	Rabbit	proteintech^TM^	14739-1-AP
AMPK	65 kd	Mouse	santa cruz	sc-25792
P-AMPKa1/2	65 kd	Rabbit	santa cruz	sc-33524
FXR	69 kd	Goat	santa cruz	sc-1205
LXR α/β	49 kd	Rabbit	santa cruz	sc-13068
PPARα	55 kd	Rabbit	santa cruz	sc-9000
GAPDH	36 kd	Mouse	KangChen Bio-tech	KC-5G4
β-actin	42 kd	Mouse	KangChen Bio-tech	KC-5A08
H1	30kd	Rabbit	Abcam	ab17584

**Table 4:** Body weight, liver weight, and serum concentrations of metabolites in weaning piglets.

Variables	Control (*n* = 8)	Betaine (*n* = 8)
Body weight, kg	7.27 ± 0.31	7.37 ± 0.40
Liver weight, g	183.7 ± 14.9	178.0 ± 10.5
Biochemical metabolites		
Serum betaine, μmol/L	1.49 ± 0.11	3.55 ± 0.31 *
TG, mmol/L	1.40 ± 0.15	1.11 ± 0.14
TCH, mmol/L	4.43 ± 0.38	4.88 ± 0.34
TBA, μmol/L	58.4 ± 4.12	4.88 ± 0.34
LDLC, mmol/L	1.94 ± 0.19	2.55 ± 0.20
HDLC, mmol/L	2.00 ± 0.14	1.81± 0.11
LDLC/ HDLC	1.00 ± 0.09	1.47 ± 0.10 *
Amino acids		
Isoleucine (μmol/L)	139 ± 13.5	148 ± 15.2
Leucine (μmol/L)	279 ± 23.8	309 ± 18.3
Lysine (μmol/L)	235 ± 10.2	259 ± 41.9
Methionine (μmol/L)	46.1 ± 6.34	82.3 ± 7.16 *
Phenylalanine (μmol/L)	90.4 ± 5.88	128 ± 10.9 *
Tyrosine (μmol/L)	299 ± 35.0	296 ± 44.5

Values are means ± SEM, *n* = 8. Different from control, * *p* < 0.05.

## Abbreviations

AHCY	S-adenosylhomocysteine hydrolase
AMPK	AMP-activated protein kinase
Bet	piglets born to sows supplemented with betaine diets
BHMT	betaine-homocysteine S-methyltransferase
BW	body weight
Con	piglets born to sows supplemented with normal diets
CYP7α1	cholesterol-7alpha-hydroxylase
CYP27α1	cholesterol-27alpha-hydroxylase
DNMTs	DNA methyltransferases
FXR	farnesoid X receptor
GNMT	glycine N-methyltransferase
H1	histone H1
H3	histone H3
H3K4me3	histone H3 lysine 4 trimethylation
H3K27me3	histone H3 lysine 27 trimethylation
HDLR	high-density lipoprotein receptor
HMGCR	3-hydroxy-3-methylglutaryl coenzyme A reductase
LDLR	low density lipoprotein receptor
LXR	liver X receptor
Met	Methionine
NR	Nuclear receptors
PPARα	peroxisome proliferator-activated receptor alpha
SAM	*S*-adenosylmethionine
SAH	*S*-adenosylhomocysteine
SETD7	SET Domain Containing Protein 7
SR-BI	scavenger receptor class B type I
Tch	total cholesterol
TBA	total bile acids
TMG	trimethylglycine
